# Supplementary material for: scGrapHiC: deep learning-based graph deconvolution for Hi-C using single cell gene expression
Source: Bioinformatics. 2024 Jun 28;40(Suppl 1):i490–500. doi: 10.1093/bioinformatics/btae223 (PMC11256916; doi:10.1093/bioinformatics/btae223)
Supplement: btae223_Supplementary_Data [file btae223_supplementary_data.zip › btae223_Supplementary_Data/Singh.292.sup.0.pdf]

Supplementary Materials for scGrapHiC: Deep learning-based graph deconvolution for Hi-C using single cell gene expression

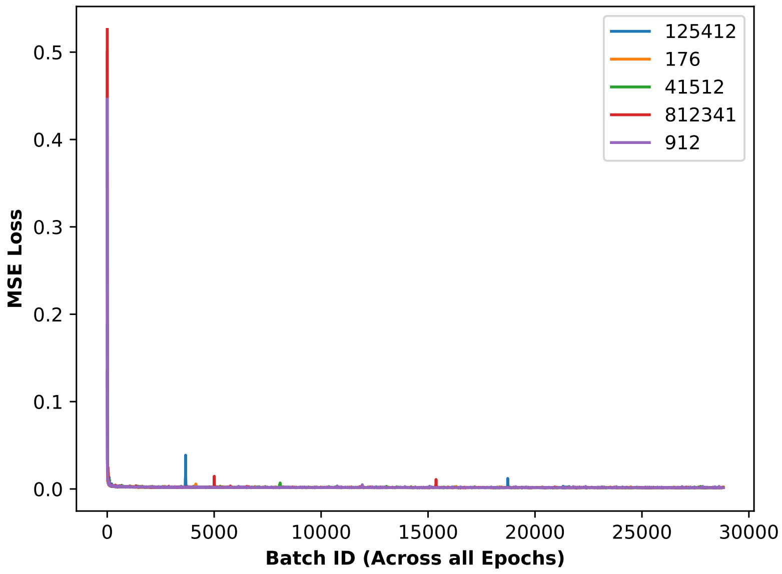

Fig. S1: We show the loss curve of scGrapHiC of 5 different random seeds and find that the model converges to a very similar loss highlighting scGrapHiC's stability to randomized weight initialization. For rest of the evaluations, we choose another random seed of 40 and show rest of the results on that seed.

	Value	Explanation	Reference
**Library Size**	25000	The library size we aim to simulate through our library size normalization pre-processing step.	Fig. S2
**Soft Threshold (t)**	1	Soft threshold value we use for smoothing the scHi-C contact matrices	Fig. S3
**Positional Encodings Dimensions (k)**	16	Top k components that we select from the eigenvectors of the bulk Hi-C as positional encodings	Fig. S4
**Supporting Features**	True	Whether we use the supporting structural features CTCF and CpG scores as additional node features	S4
**Number of cells cutoff**	190	We filter our cell-types during pseudo-bulk that have less than 190 cells.	Fig. S5, S6

Table S1. This table mentions all the hyperparameters, what their purpose is and refers to the appropriate figure/table explaining the tuning process.

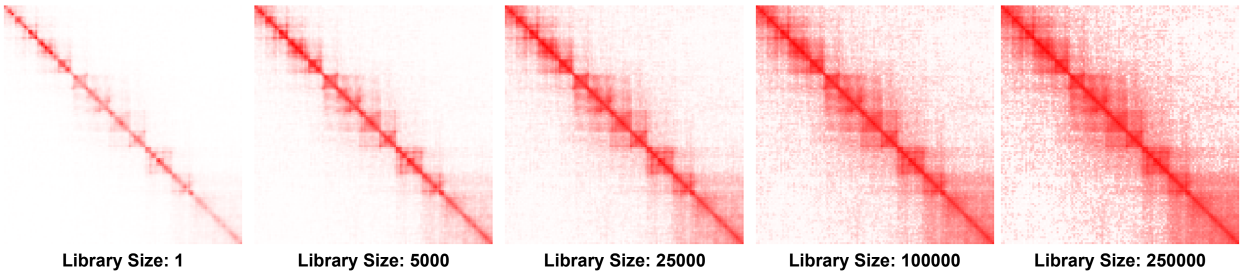

Fig. S2: Tuning of the library size parameter, we that using a value of 25000 allowed us to enhance the structure while keeping the background noise low.

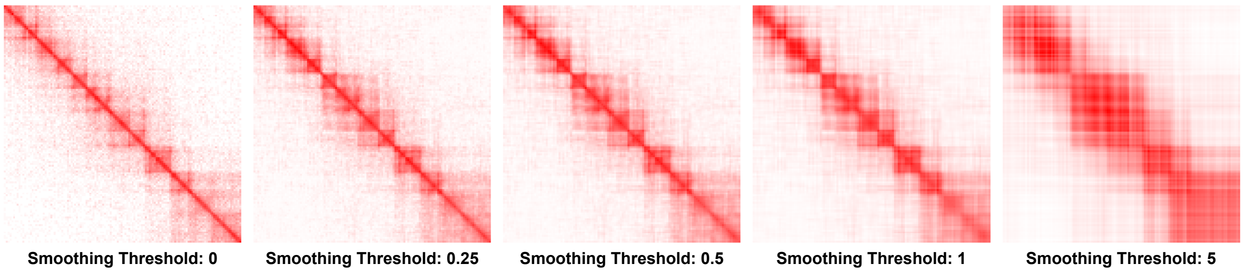

Fig. S3: Tuning of the Hi-C smoothing soft thresholding parameter, and we found that using 0.25 ensures that we are able to supress the background noise while preserving the hierarchical organization of the genome.

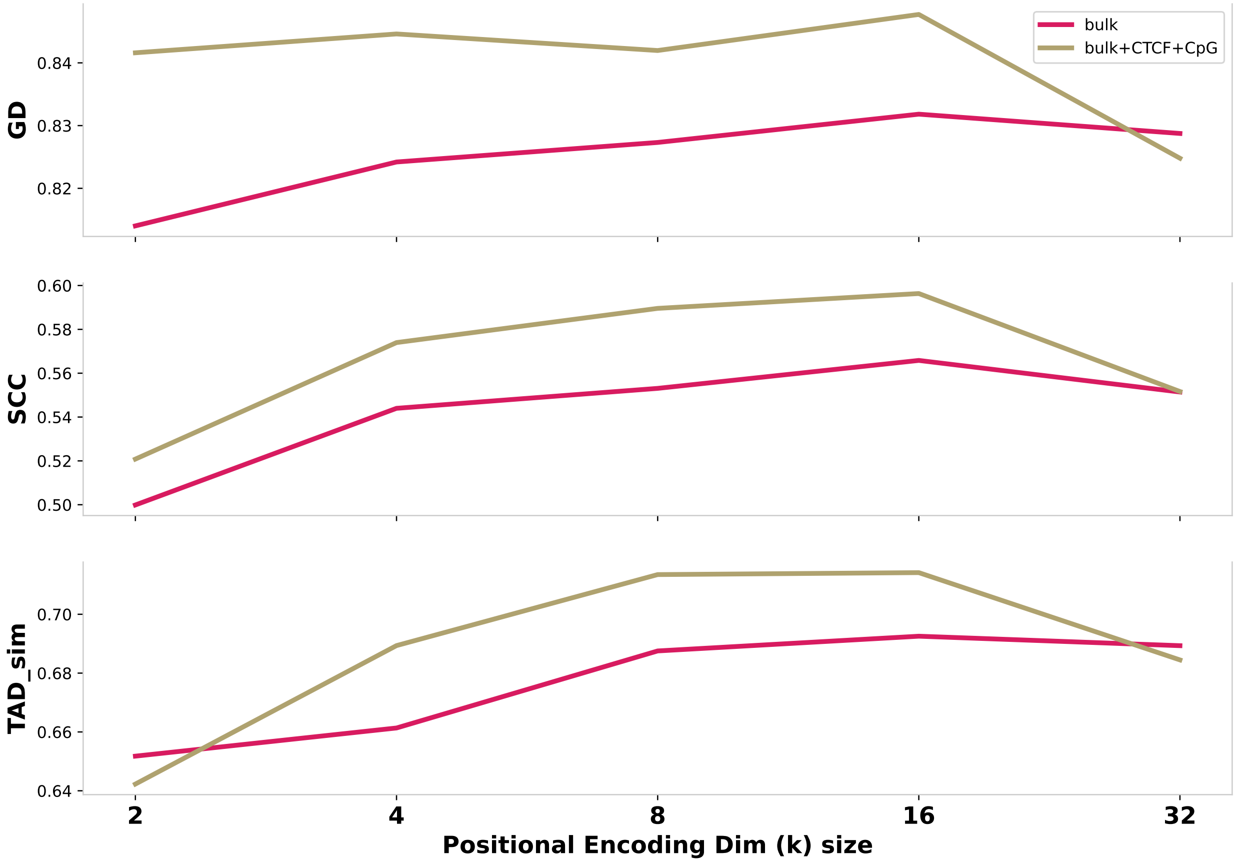

Fig. S4: Positional encoding vector of size 16 found through hyperparameter tuning on different values of k

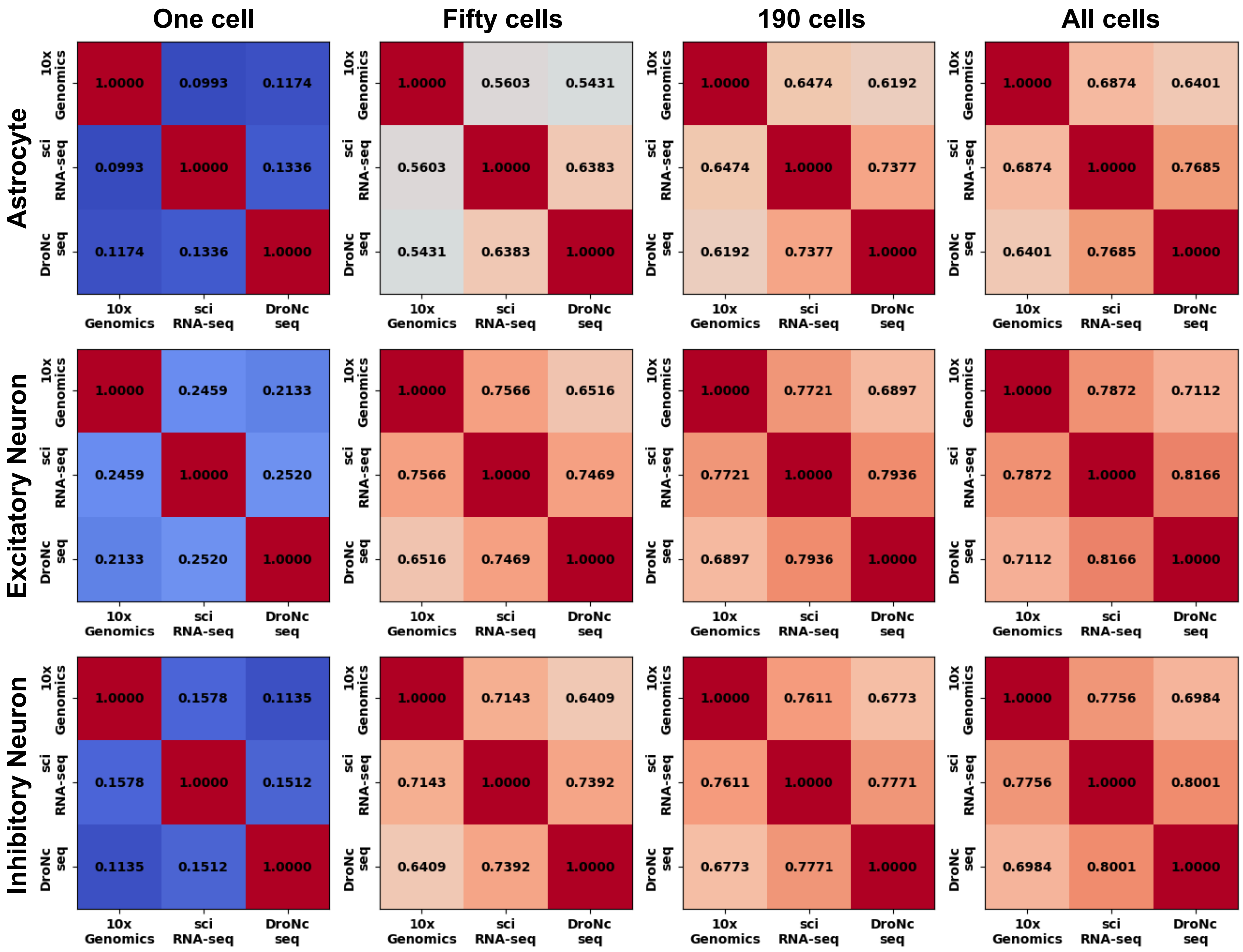

Fig. S5: This analysis highlights that pseudo-bulking suppresses the batch effects originating in single-cell datasets generated through various sequencing protocols. We analyzed the Mouse cortex data we acquired from GEO GSE132044, which contains scRNA-seq data on the Mouse cortex sample originating from 10x Genomics, sci-RNA-seq, and DroNc-Seq platforms. We find that pseudo-bulking across Astrocytes, Inhibitory, and Excitatory neurons improves Pearson's correlation between the scRNA-seq profiles of the same cells originating from different experiments. We achieved similar correlation scores at the 190 cell cut-off as if we were using all the cells.

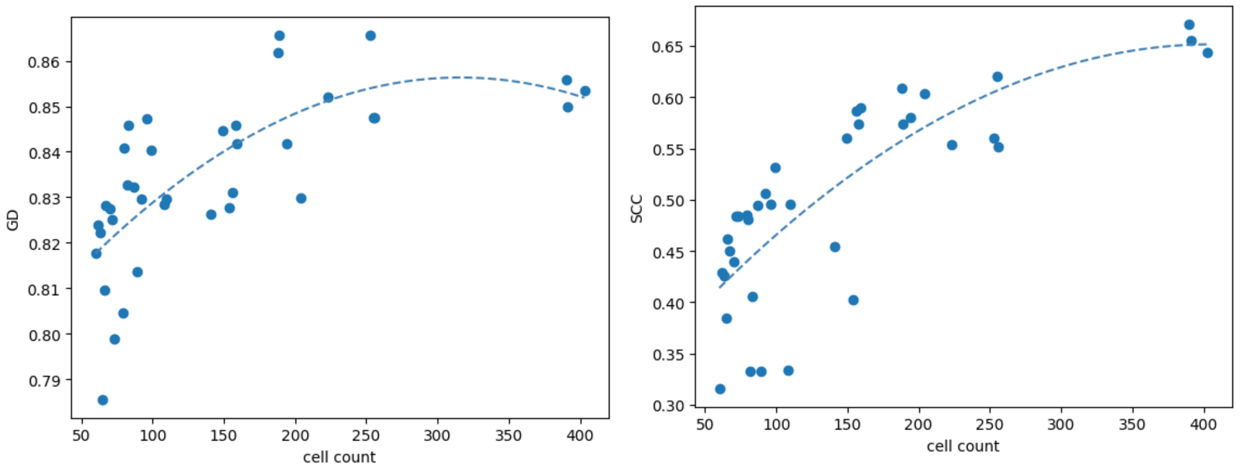

Fig. S6: Adjusting for the pseudo-bulk number of cells cutoff parameter shows that the performance degrades non-linearly as the number of cells per pseudo-bulk go down. Suggesting a drastic loss of structure and cell-identifying features making it challenging for the current setup to reliably predict scHi-C contact maps.

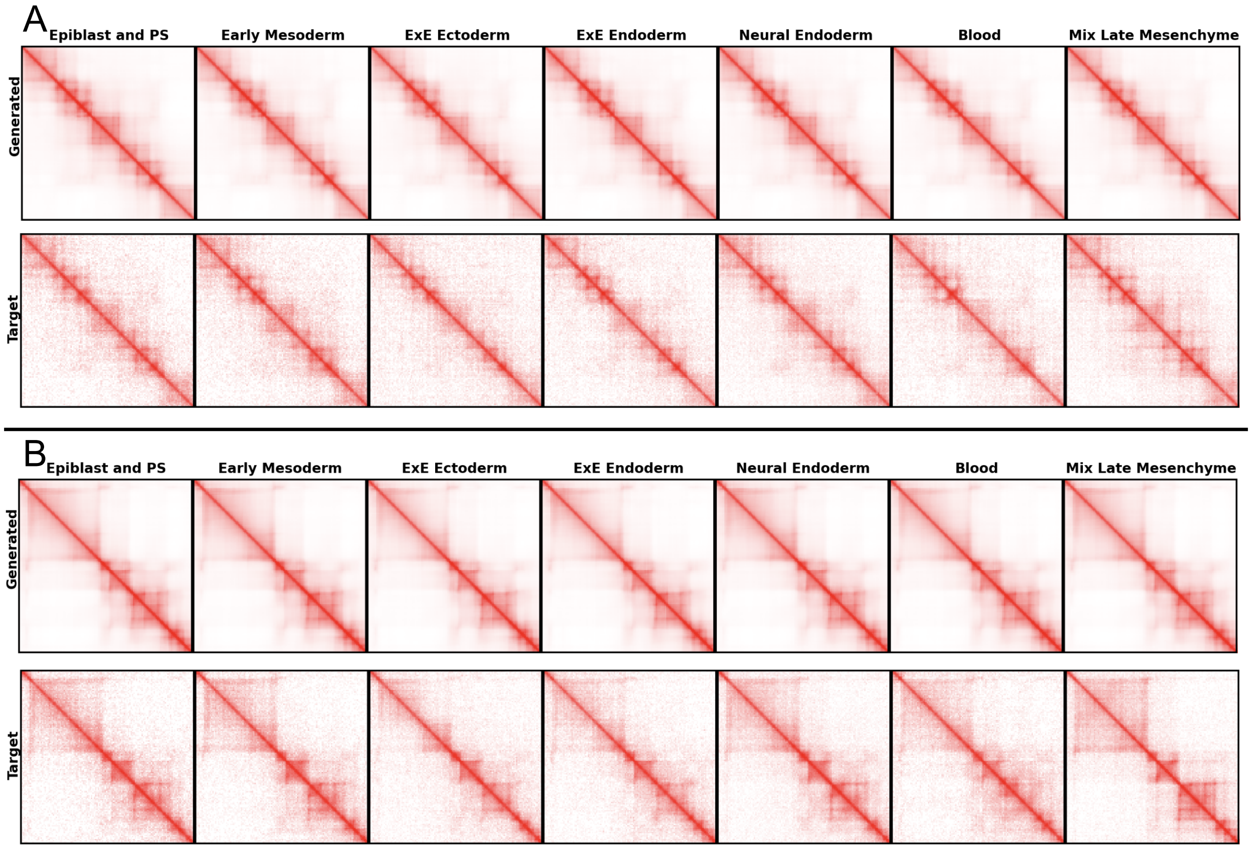

Fig. S7: We show two different regions from Chr 7 in **A** and Chr 11 in **B**. We find that scGrapHiC is able to accurately infer complex chromatin hirearchical strucures accurately. However, in **B**, we also find that scGrapHiC mispredicts the presence of a sub-TAD structure in blood cell-type which we believe can be attributed to sparse coverage either in the scRNA-seq or scHi-C or both for this cell-type.

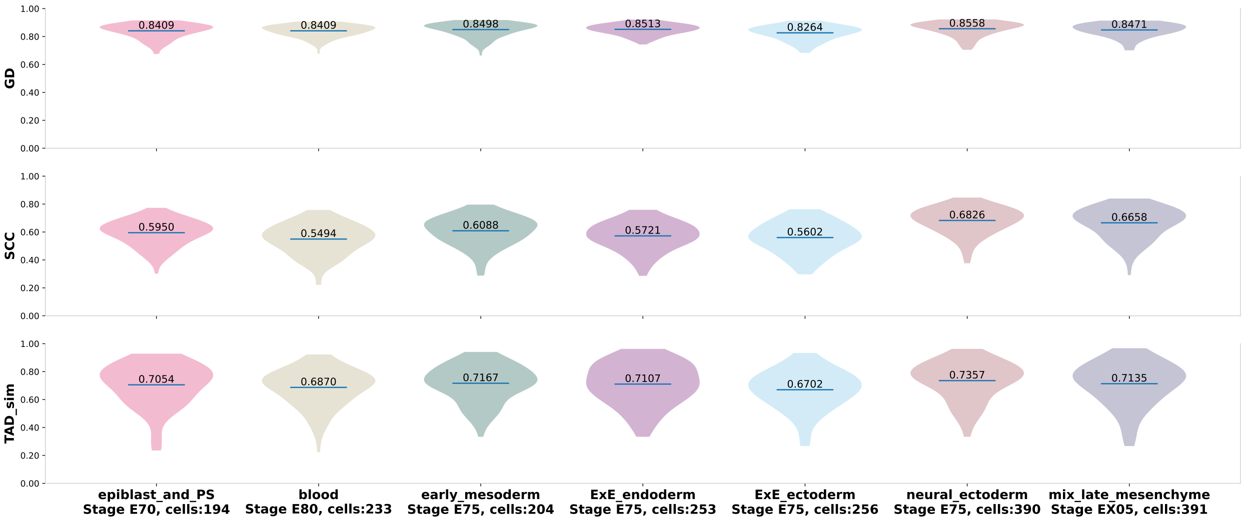

Fig. S8: We show the scores on each cell-type individually across GD, SCC and TAD Sim metrics. We find that the scores stay fairly consistent however, the cells with lower coverage tends to get less scores in all three metric. This correlation highlights the inherent limitation of working with sparse single cell datasets.
